# Merlin Is a Negative Regulator of Human Melanoma Growth

**DOI:** 10.1371/journal.pone.0043295

**Published:** 2012-08-17

**Authors:** Lucas B. Murray, Ying-Ka Ingar Lau, Qin Yu

**Affiliations:** Department of Oncological Sciences, Mount Sinai School of Medicine, New York, New York, United States of America; The Moffitt Cancer Center & Research Institute, United States of America

## Abstract

Merlin is encoded by the neurofibromatosis type 2 (*NF2*) gene and is a member of the Band 4.1 protein family. This protein acts as a linker that connects cell surface proteins to the actin cytoskeleton. Defects caused by mutations of the *NF2* gene give rise to NF2 disease, which is generally characterized by the formation of bilateral vestibular schwannomas and, to a lesser extent, meningiomas and ependymomas. In addition to these tumor types, *NF2* is mutated and/or merlin expression is reduced or lost in numerous non-NF2 associated tumors, including melanoma. However, the role of merlin in human melanoma growth and the mechanism underlying its effect are currently unknown. In the present study, we show that merlin knockdown enhances melanoma cell proliferation, migration, and invasion *in vitro* and that decreased merlin expression promotes subcutaneous melanoma growth in immunocompromised mice. Concordantly, we find that increased expression of merlin in a metastatic melanoma cell line reduced their *in vitro* migration and proliferation, and diminished their ability to grow in an anchorage independent manner. Increased merlin expression also inhibits *in vivo* growth of these melanoma cells. Lastly, we demonstrate that higher merlin levels in human melanoma cells promote the H_2_O_2_-induced activation of MST1/2 Ser/Thr kinases, which are known tumor suppressors in the Hippo signaling pathway. Taken together, these results provide for the first time evidence that merlin negatively regulates human melanoma growth, and that loss of merlin, or impaired merlin function, results in an opposite effect. In addition, we show that increased merlin expression leads to enhanced activation of the MTS1/2 kinases, implying the potential roles of MST1/2 in mediating the anti-melanoma effects of merlin.

## Introduction

Melanoma is the deadliest form of skin cancer. It is readily curable if diagnosed at an early stage, however a large percentage of melanomas arise without association with premalignant nevi [1]. This leads to ineffective early detection and results in approximately ten percent of patients presenting with metastatic disease upon first diagnosis [2]. The current standard of care for advanced melanoma has a response rate of less than 20% [3], [4] and the median survival length for these patients is less than one year [5]. However, treatment options have recently been substantially improved due to the discovery and successful clinical trials of an inhibitor of mutated BRAF (BRAF^V600E^), vemurafenib (PLX4032) [6]. Approximately 50% of malignant melanomas harbor a BRAF activating mutation, the majority of these being BRAF^V600E^ [7], [8], which results in constitutive activation of BRAF and increased activation of the MAP kinase pathway. However, nearly 50% of melanomas do not harbor a BRAF^V600E^ mutation and do not respond to these specific inhibitors. Furthermore, there is anticipated development of acquired resistance to the BRAF inhibitors [9]. Thus, it is essential to identify additional signaling pathways and molecules that play critical roles in melanoma growth and progression, which could serve as the potential points of intervention for future therapies.

Merlin (**m**oesin-**e**zrin-**r**adixin **l**ike prote**in**) isoform I is a 595 amino acid protein encoded by the *NF2* gene that shares significant sequence similarities with the ezrin, radixin, moesin (ERM) family of cytoskeletal linker proteins [10], [11]. Mutations of this gene result in Neurofibromatosis type 2 (NF2), a dominantly inherited cancer syndrome. NF2 patients are born with a mutant allele of *NF2* and, upon loss of heterozygosity, develop tumors of the central and peripheral nervous system. The resulting tumors are predominantly benign schwannomas, meningiomas, and ependymomas [12]. Sporadic NF2-associated tumor types have also been shown to arise concurrently with the loss of merlin expression [13]. The tumors resulting from loss of merlin function may be attributed to the loss of contact inhibition of cell growth that is known to be maintained by merlin in their non-transformed progenitors [14].

Merlin has been implicated in a variety of signaling cascades. Due to its proximal location to the cell membrane, merlin is in a position to modulate numerous cell-cell and cell-matrix interactions [15]. Merlin negatively regulates several different receptor tyrosine kinases (RTK’s) including epidermal growth receptor (EGFR) [16], platelet-derived growth factor (PDGF) [17], ErbB2 [18], and insulin-like growth factor receptor [19]. CD44 function has also been shown to be inhibited by merlin, which mediates, in part, the tumor suppressor activity of merlin [15], [20], [21], [22]. Merlin also modulates the activity of non-receptor Thr/Ser kinases such as PAK1/2 [23]. Additionally, merlin regulates cell proliferation and differentiation [24], and functions upstream of the Hippo signaling pathway [15], [25], [26], [27], [28]. However, it remains unclear as to how merlin exerts its tumor suppressor function in mammalian cells and how it contributes to cancer growth and progression.

Recently, the roles of merlin have begun to expand beyond the NF2 associated tumors. Even in the absence of genetic alterations, merlin levels have been shown to be down regulated, or its activation inhibited, in mesothelioma [29], glioma [25], prostate [30], and breast cancer [31]; and merlin has been shown to play significant roles in inhibiting the progression of these cancer types [25], [29], [30], [31]. Early studies have also revealed a low frequency of *NF2* mutations in approximately 5% of observed human melanoma cases (www.sangerinstitute.com) [32]. Among these mutated cases, ∼17% (1/6) and ∼83% (5/6) were from superficial spreading melanoma and metastatic melanoma, respectively. This fact, coupled with the emerging role of merlin in the non-NF2 associated tumors, led us to investigate the role for merlin in human melanoma growth. We show that reduced merlin protein level leads to enhanced melanoma cell proliferation, migration, and invasion *in vitro*. On the contrary, merlin overexpression leads to reduced melanoma cell proliferation and migration, as well as reduced anchorage independent growth. We demonstrate that merlin knockdown promotes subcutaneous growth of WM1552C human melanoma cells, whereas merlin overexpression inhibits MeWo melanoma growth *in vivo*. Furthermore, we find that the established WM1552C tumors derived from the merlin-positive parental cells had lost expression of endogenous merlin, suggesting that selective loss of merlin promotes melanoma growth *in vivo*. This notion was further verified by our finding that merlin knockdown confers tumorigenicity to a non-tumorigenic melanoma cell line. Lastly, we establish that increased merlin expression leads to enhanced activation of MST1/2 kinases, the mammalian homologues of *Drosophila* Hippo, which are established tumor suppressors and key regulators of cell proliferation and apoptosis. These data show for the first time that merlin inhibits human melanoma growth and enhances activation of MST1/2 in human melanoma cells and that merlin may exert its anti-melanoma activity through regulating MST1/2 activity.

## Results

### Merlin Knockdown Promotes Proliferation, Motility, and Invasiveness of Human Melanoma Cells

**Figure 1:**
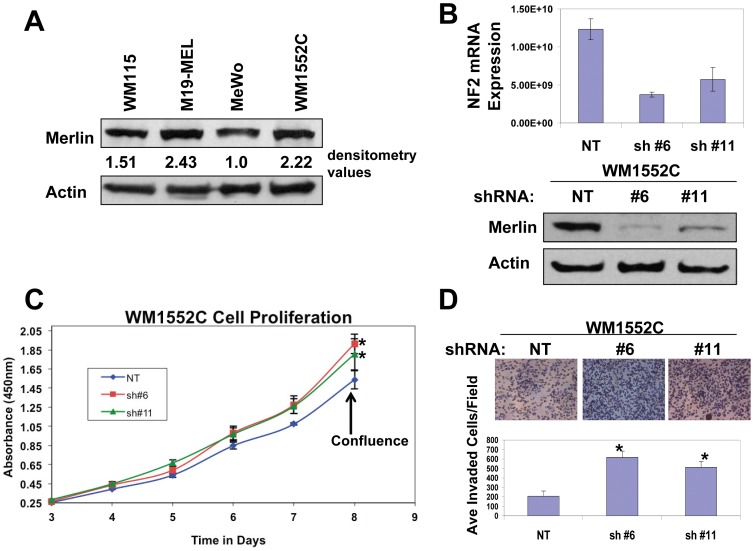
Merlin knockdown promotes proliferation and invasiveness of WM1552C human melanoma cells. **A**, Western blot analysis of endogenous merlin expression in human melanoma cell lines. Actin was used as a loading control. 50 µg of protein were loaded in each lane. Numbers are densitometry values used to normalize merlin expression to actin loading controls. **B**, Top Panel, Real-time-RT-PCR to assess the merlin mRNA levels in WM1552C cells transduced with non-targeting (NT) or merlin shRNA (#6 and #11). Bottom Panel, Western blot analysis of merlin protein levels in WM1552C cells transduced with the non targeting shRNA (NT) or shRNA targeting human NF2 (sh#6 and sh#11). Actin was used as a loading control. 50 µg of protein were loaded in each lane. **C**, WST-1 Proliferation assays of the transduced WM1552C cells. *denotes a *p*-value <0.05. **D**, Matrigel invasion assays of the transduced WM1552C cells. Bars represent the mean invaded cells in 15 randomly selected 200X microscopic fields +/− SD. *denotes a *p*-value <0.01.

In order to determine how merlin affects melanoma cell behavior, we first assessed the endogenous levels of merlin protein in four human melanoma cell lines. Upon normalization to actin levels, we identified that WM1552C cells express a relatively high endogenous level of merlin (Figure 1A). This, coupled with the fact that WM1552C cells were originally isolated from a primary melanoma lesion at the transition between radial growth phase (RGP) and vertical growth phase (VGP) (www.atcc), provided us with a cell line to explore the effects of merlin expression on tumorigenicity.

**Figure 2:**
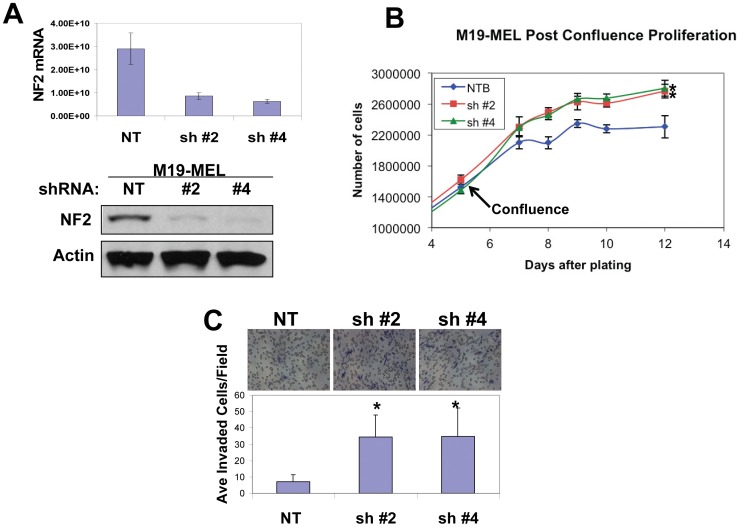
Merlin knockdown promotes proliferation and invasiveness of M19-MEL human melanoma cells. **A**, Top Panel, q-real-time RT-PCR was performed to assess merlin mRNA levels in M19-MEL cells transduced with non-targeting (NT) or merlin shRNA (#2 and #4). Bottom Panel, Western blot analysis of merlin protein levels in M19-MEL cells transduced with non targeting shRNA (NT) or shRNA targeting NF2 (sh#2 and sh#4), actin was used as a loading control. 50 µg of protein were loaded in each lane. **B**, Proliferation assay of the transduced M19-MEL cells. *denotes a *p*-value <0.05. **C**, Matrigel invasion assay of the transduced M19-MEL cells: Bars represent the mean invaded cells in 30 randomly selected 200X microscopic fields. *denotes a *p*-value <0.01.

To assess the effects of reduced merlin expression on this early stage melanoma, we screened a set of shRNAs against human merlin (Open Biosystems) and found that shRNAs #6 and #11 (sh#6 and sh#11), but not a non-targeting shRNA control (sh NT), effectively knocked down merlin expression in WM1552C cells at both the mRNA and protein level (Figure 1B). We then used these transduced cells to investigate how reduced merlin expression affects their *in vitro* proliferation. We found that merlin knockdown promoted post-confluence cell proliferation (Figure 1C), which is consistent with the reported role of merlin in maintenance of the contact inhibition of cell growth [14]. This result was validated in a second melanoma cell line, M19-MEL, which expresses a comparable endogenous level of merlin (Figure 2A–B).

In addition to proliferation, merlin has been implicated in the regulation of several other tumorigenic characteristics, such as cell migration and invasion, which led us to investigate the merlin effects on motility and invasiveness of these melanoma cells. Indeed, reduced merlin levels significantly enhanced motility and invasiveness of WM1552C cells (Figure S1A, Figure 1D). These effects were validated in the M19-MEL melanoma cell line (Figure S1B and Figure 2C). Taken together, these results suggest that merlin inhibits human melanoma cell proliferation, migration, and invasion.

### Merlin Knockdown Promotes Subcutaneous Growth of WM1552C Human Melanoma Cells

**Figure 3:**
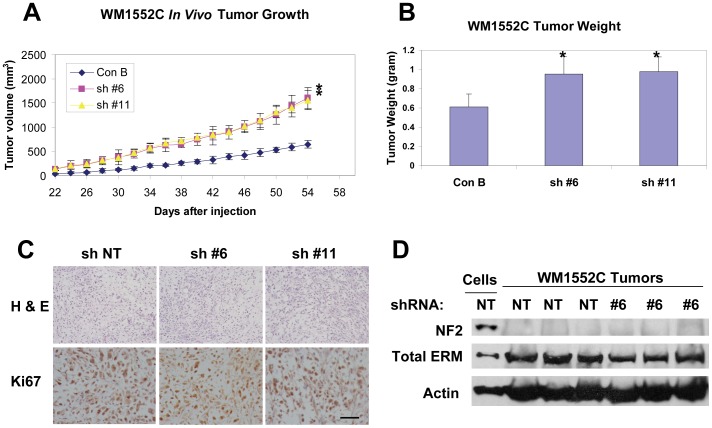
Merlin knockdown enhances in vivo tumor growth of WM1552C human melanoma cells. **A**, Growth curves of the subcutaneous tumors derived from 10×10^6^ injected cells of indicated transduction. Numbers are mean tumor volumes (mm^3^) +/− SD. * =  *p*-value <0.05, n = 8. **B**, Tumor weights at the completion of the subcutaneous tumor experiments. Bars represent mean +/− SD tumor weights of n = 8 mice/group. *denotes *p*-value <0.05. **C**, H & E staining and Ki67 IHC staining of the subcutaneous melanomas isolated at 54 days post injection of the tumor cells into immunocompromised Rag2/II2rg mice. Bar, 80 µm. **D**, Western blot analysis of merlin and total ERM protein levels in WM1552C cells transduced with non-targeting shRNA (Lane 1) or in the WM1152C tumor lysates derived from the indicated transduced cells (Lanes 2–7). 50 µg of protein was loaded into each lane and actin was included as a loading control.

To determine the effect of merlin knockdown on melanoma cell growth *in vivo*, the transduced WM1552C cells were used to assess subcutaneous growth rates. We found that merlin knockdown promotes the subcutaneous growth of WM1552C cells (Figure 3A) and leads to significantly increased tumor weights at the end of a 54 day *in vivo* study (Figure 3B). To investigate a possible underlying cellular mechanism, the tumor sections were stained for Ki67, a marker of cell proliferation. Surprisingly, there was little difference between the numbers of Ki67 positive cells in these tumor sections derived from the implanted melanoma cells with or without merlin knockdown (Figure 3C). To understand the basis of this apparent lack of differences in the *in vivo* proliferation rates in these established melanomas, we assessed merlin protein levels in the tumor lysates derived from these *in vivo* experiments. We found that there were low levels of merlin in these established tumors regardless of whether they were derived from the transduced WM1552C cells originally expressing merlin or not (Figure 3D). On the contrary, total ERM proteins are readily detectable in all of these tumor lysates (Figure 3D). Furthermore, WM1552C tumor sections derived from these *in vivo* experiments displayed similarly low merlin levels whereas there are readily detectable levels of merlin in the control sections derived from human nevi and MeWo tumors expressing exogenous merlin (Figure S2). These results provided strong support that there is a selective down regulation of merlin expression in the established melanoma tumors regardless of the original merlin levels in the melanoma cells. Consistent with these findings, merlin knockdown in M19-MEL human melanoma cells conferred *in vivo* tumorigenicity (Table S1). Taken together, these results strongly suggest that merlin negatively regulates human melanoma growth, whereas loss of merlin results in an opposite effect.

### Elevated Merlin Levels Inhibit the Tumorigenicity of MeWo Human Melanoma Cells

**Figure 4:**
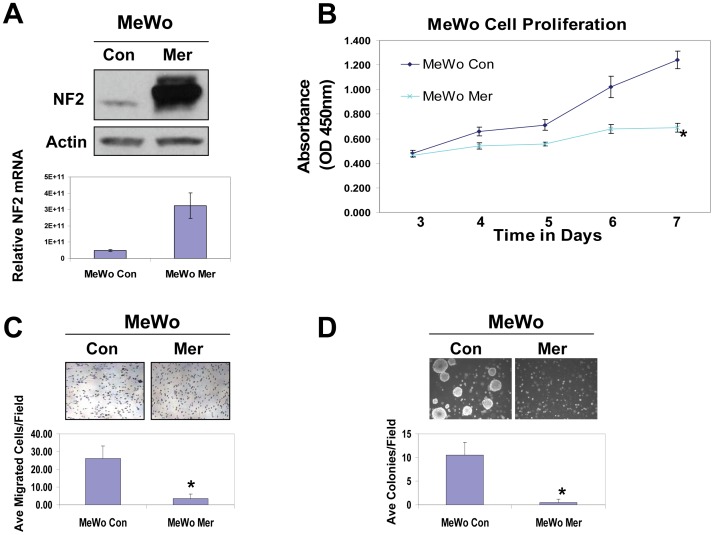
Increased merlin levels inhibit proliferation, migration and anchorage-independent growth of MeWo human melanoma cells. **A**, Top panel: Western blot of MeWo cells overexpressing merlin (MeWo Mer) or transduced with an empty vector control (MeWo Con). 50 µg of protein was loaded into each lane. Actin was included as a loading control. Bottom Panel: NF2 mRNA levels in the transduced MeWo cells were determined by quantitative Real-time RT-PCR. **B**, WST-1 proliferation assay of the cells transduced with merlin or the empty vector control. **C**, Transwell migration assay demonstrates that merlin significantly reduces the motility of MeWo cells. Bars represent the mean numbers of cells migrated through transwells in 15 randomly selected 200X microscopic fields. *denotes a *p*-value <0.01. **D**, MeWo cells transduced with merlin display an impaired ability to form colonies in soft agar. Graph represents the mean number of colonies in 15 randomly selected microscopic fields. *denotes *p*-value<0.05.

Further screening of an additional 10 human melanoma cell lines has revealed a subset of metastatic cell lines that express little or significantly reduced merlin protein (Figure S3, arrows). One of these human metastatic melanoma cell lines with relatively low merlin expression is MeWo. In order to confirm the effects of merlin knockdown on human melanoma, we used a reciprocal strategy by overexpressing merlin in this metastatic MeWo melanoma cell line (MeWo Mer). Stably transduced pooled populations of MeWo Mer cells expressed approximately 6 fold higher merlin transcripts when compared to the MeWo control cells (MeWo Con) transduced with the empty expression vector alone (Figure 4A). When compared to MeWo Con cells, overexpression of merlin led to a significant inhibition of *in vitro* cell proliferation (Figure 4B). Elevated merlin levels also caused a decrease in tumor cell migration (Figure 4C) and dramatically inhibited their ability to grow in an anchorage independent manner (Figure 4D). Consistent with our knockdown data, these results further established that merlin plays inhibitory roles in many aspects of tumorigenicity of human melanoma cells.

### Increased Merlin Levels Reduce in vivo Melanoma Proliferation and Invasiveness

**Figure 5:**
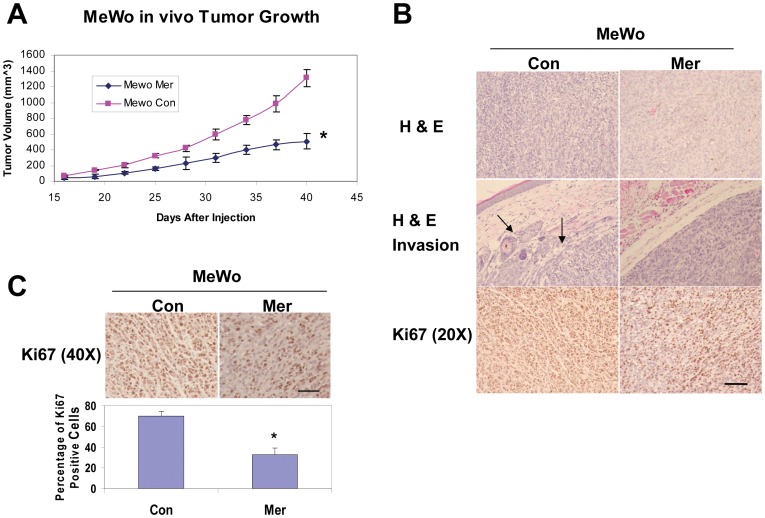
Increased merlin levels inhibit in vivo melanoma growth and invasion. **A**, Subcutaneous growth rates of the melanomas derived from 1×10^6^ transduced MeWo cells as indicated. Numbers are the mean tumor volumes (mm3) +/− SD. * =  *p*-value <0.05, n = 6. **B**, H&E and IHC staining with an anti-Ki67 antibody of the tumor sections derived from MeWo Mer or MeWo Con cells. Bar, 200 µm. **C**, Quantification of the percentage of Ki67 positive cells in the tumor sections derived from MeWo Mer and MeWo Con cells. Bars represent the means of 15 randomly selected 40X microscopic fields. *denotes a *p*-value <0.05. Bar, 200 µm.

To assess the effects of elevated merlin level on *in vivo* melanoma formation, the transduced MeWo cells were injected subcutaneously into immunocompromised mice. MeWo cells overexpressing merlin grew at a rate significantly slower than cells transduced with the empty expression vector (Figure 5A). To understand the cellular mechanism behind the anti-melanoma effect of merlin, we assessed the effect of merlin on melanoma cell proliferation by staining the tumor sections with an anti-Ki67 antibody. When compared to the tumors derived from MeWo Con cells, we found that the tumor sections derived from MeWo Mer cells displayed significantly decreased percentages of Ki67 positive cells, and therefore decreased rates of proliferation (Figure 5C). In addition, the tumors derived from MeWo Mer cells appeared to have well defined tumor borders, whereas the tumors derived from MeWo Con cells displayed regions with less defined tumor borders where the tumor cells were migrating/invading into adjacent host tissues, indicating a more invasive phenotype (Figure 5B, arrows). Together, these results support the notion that merlin inhibits melanoma tumor growth/proliferation as well as their invasiveness *in vivo*.

### Increased Merlin Levels Lead to the Enhanced Activation of MST1/2 Kinases Induced by Stress

**Figure 6:**
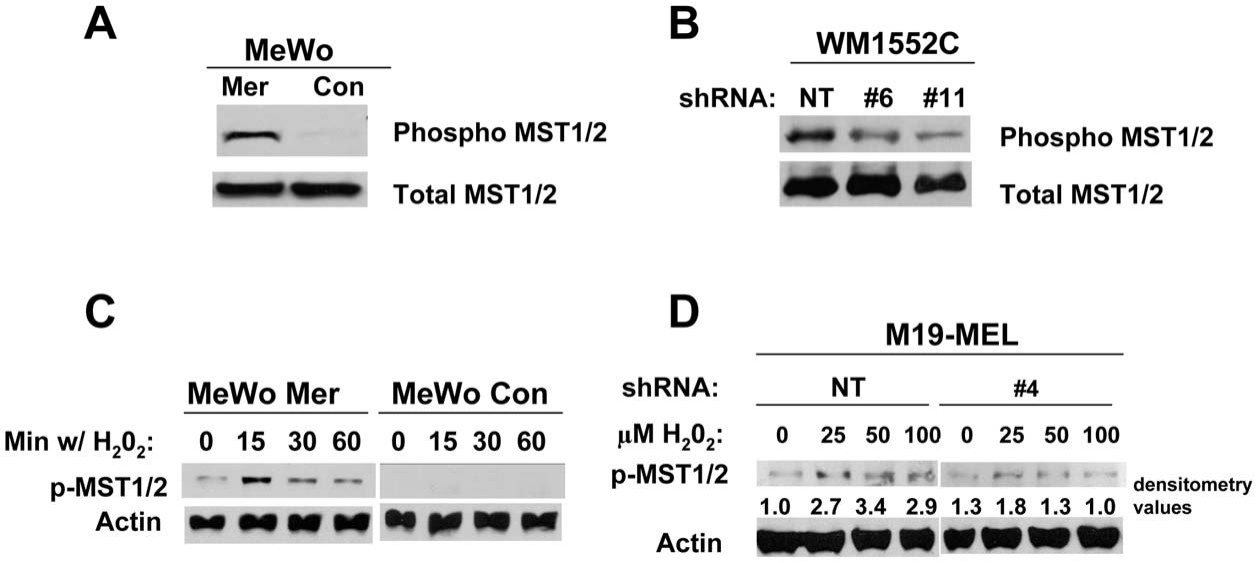
Increased merlin levels promote MST1/2 kinase activation whereas merlin knockdown impairs the stress-induced activation of MST1/2. **A**, Western blot analysis of phosphorylated MST1/2 kinases was performed in the transduced MeWo cells at 80% confluence. **B**, Western blot analysis of phosphorylated MST1/2 kinases in the confluent WM1552C cells with or without merlin knockdown. **C**, Western blot analyses of the transduced MeWo cells treated with H_2_O_2_ and probed for activation of MST1/2 Ser/Thr kinases. The cells were treated with 500 µm H_2_O_2_ for indicated times. **D**, Western blot of the cell lysates derived from transduced M19-MEL cells treated with indicated H_2_O_2_ concentrations for 15 min. 50 µg of protein was loaded in each lane and actin was included as a loading control in panels C and D. Numbers are densitometry values used to normalize phosphor-MST1/2 to actin loading controls.

To determine the molecular mechanism underlying the anti-melanoma effects of merlin, we assessed its effects on activation of several signaling molecules, including those in the Hippo signaling pathway. We found that MeWo Mer had a higher basal level of active MST1/2 (phospho-MST1/2) when compared to MeWo Con cells (Figure 6A). In addition, upon reaching confluence, WM1552C cells with merlin knocked down displayed a lower level of active MST1/2 kinases compared to the WM1552C cells without merlin knockdown (Figure 6B). MST1/2 kinases are activated by a variety of stresses including hydrogen peroxide (H_2_O_2_) [33]. To determine whether merlin affects the stress-induced activation of these kinases, MeWo cells with or without merlin overexpression were treated with sub-lethal amounts of H_2_O_2_. We found that increased expression of merlin promotes the H_2_O_2_-induced activation of MST1/2 kinases (Figure 6C). We confirmed this result and showed that merlin knockdown in M19-MEL cells impaired the H_2_O_2_-induced activation of MST1/2 kinases (Figure 6D). Together, these results provided strong evidence that increased merlin levels enhance basal activation as well as the stress-induced activation of MST1/2 in human melanoma cells.

## Discussion

Recent studies have suggested that merlin may serve as an important inhibitor of a variety of non-NF2 associated tumors [25], [29], [30], [31]. The goal of this study was to determine the effect of merlin on human melanoma growth and establish the underlying mechanism. We show that merlin knockdown, in two independent melanoma cell lines, led to increased post-confluent proliferation *in vitro,* whereas elevated merlin levels decreased the proliferation of a metastatic melanoma cell line (MeWo) even before the cells reached confluence. This confluence-independent effect of merlin on MeWo melanoma cell proliferation may be due to the much higher merlin protein levels in MeWo Mer cells compared to endogenous merlin levels in WM1152C cells. We have shown that the higher merlin expression in MeWo Mer cells resulted in increased basal activity of MST1/2 kinases (Fig. 6A), which may mediate the confluent independent proliferative effect in the presence of a higher level of merlin, whereas WM1552C cells with merlin knockdown display an impaired ability to activate MST1/2 only after the cells reach confluence (Figure 6B).

Our studies also established that merlin inhibited melanoma cell motility and invasiveness *in vitro* and *in vivo*. These results are consistent with a recent finding that elevated merlin levels decrease the metastatic potential of B16 murine melanoma cells [34]. The exact mechanism of this merlin effect remains to be determined, however, merlin has been shown to regulate key components of actin cytoskeleton dynamics, including PAK1/2 kinases and the activities of small GTPases such as Rac, all of which play essential roles in cell motility and invasiveness.

In addition to invasion/migration and *in vitro* proliferation effects, we demonstrate that elevated merlin levels decrease *in vivo* melanoma growth whereas merlin knockdown significantly enhances tumor growth. Merlin knockdown results in a more dramatic pro-melanoma growth effect *in vivo* than the observed pro-proliferation effect *in vitro*. We first assessed the possibility that the FBS in the cell culture media may affect endogenous merlin expression, which could be partially responsible for this difference. To assess the potential effect of FBS levels on merlin expression, we cultured WM1552C cells in MCDB media with 2% or 10% FBS, and in RPMI media with 10% FBS. As shown in Figure S4 the change in FBS concentration did not alter the expression level of merlin in WM1552C cells. We believe that the differences in merlin effects *in vitro* and *in vivo* cannot be accounted for solely by the autocrine anti-proliferative effect of merlin. As we have shown that merlin functions upstream of the Hippo signaling pathway and reduced merlin expression impairs the stress-induced activation the pro-apoptotic kinases MST1/2 (Figure 6) [25]. It is likely that knockdown of merlin not only promotes melanoma cell proliferation but also reduces activation of MST1/2 kinases/Hippo signaling induced by the stresses derived from the tumor microenvironment and therefore, melanomas with reduced merlin levels are more resistant to apoptosis induced by these stresses. In addition, the downstream effectors of merlin, the components of the Hippo signaling pathway, are known to have non-cell-autonomous effects [35], which can only manifest fully *in vivo* where melanomas engage in cross-talk with their microenvironment.

To determine the underlying mechanism of the anti-melanoma effect of merlin, we explored the involvement of the Hippo signaling pathway. We established that increased merlin level enhances activation of the MST1/2 ser/thr kinases in human melanoma whereas merlin knockdown impairs the H_2_O_2_ induced activation of MST1/2. MST1/2 kinases have recently begun to emerge as putative tumor suppressors in hepatocellular carcinoma (HCC) [36], [37], [38] and as established tumor suppressors in other cancer types [39], [40]. These kinases are reported to target a number of pathways in order to exert their anti-proliferation effect and to promote apoptosis. MST1/2 kinases have been implicated in the activation of the LATS1/2 tumor suppressor kinases [41] and in subsequent inhibition of the downstream oncogenic co-transcription factor YAP1 [42], [43]. Also, MST1/2 kinases have been shown to directly phosphorylate FOXO3 [44] and activate JNK [45], all of which promote apoptosis. However, our results suggested that merlin mediated activation of MST1/2 did not lead to activation of the above signaling components (data not shown), implying that other, yet to be identified, downstream signaling components of MST1/2 may mediate the anti-melanoma effects of merlin.

Late stage melanoma is notoriously resistant to therapeutic interventions. To achieve better clinical outcomes for patients, we need to identify novel signaling pathways and components that play essential roles in melanoma progression. Here, we show for the first time that increased merlin levels sensitize melanoma to the stress induced activation of MST1/2 tumor suppressors, thus identifying two novel key signaling components that are important for melanoma growth and for its response to cellular stresses. Although merlin itself is an undruggable target, its levels and activity can be modulated through a variety of post-translational modifications. Phosphorylation at Ser518 of merlin inactivates its growth inhibitive activity. This phosphorylation can be achieved by cyclic AMP-dependent protein kinase A (PKA) [46] and p21-activated kinases 1 (PAK1) [47]. Phosphorylation of merlin by Akt at Thr230 and Ser315 can also target it for ubiquitination and subsequent degradation [48], which is a mechanism known to down-regulate merlin protein expression in breast cancer [31]. The PI3K/Akt pathway is dysregulated in a large percentage of human melanoma [49] and Akt is an established target for combination therapies in melanoma. Akt not only targets merlin for degradation, but also directly negatively regulates MST1/2 activity. Therefore, targeting AKT may lead to increased levels/activity of merlin and its downstream signaling components, thereby achieving the anti-tumor effects and sensitizing the response of melanomas to cytotoxic insults. Further investigation is required to determine how these new findings can be used to better target melanomas and design more efficacious combination therapies.

## Materials and Methods

### Cell Culture

M19-MEL and MeWo cells were obtained from NCI and ATCC, respectively, and grown in RPMI media supplemented with 10% FBS and penicillin/streptomycin. WM115 and WM1552C cell lines were obtained from ATCC and were deposited by Dr. Meenhard Herlyn as a part of the Wistar Special Collection [50]. WM1552C and WM115 cells were grown in MCDB135 media supplemented with 2%FBS, insulin, CaCl_2_, and penicillin/streptomycin. All cells were cultured in a 5% CO_2_ humidified incubator. Transduced cells were cultured in the same manner with the addition of puromycin and/or G418 to their respective media.

MeWo cells were derived from malignant human melanoma cells that metastasized to the lymph node (www.atcc.org). WM115 cells were derived from a primary human melanoma and WM1552C cells were originally isolated from a primary human melanoma lesion at the transition between radial growth phase (RGP) and vertical growth phase (VGP) (www.atcc.org) [50]. M19-MEL cells were derived from an amelanotic melanoma (http://dtp.nci.nih.gov/branches/btb/tumor-catalog.pdf).

### Western Blot

Cells were lysed in 4X SDS sample buffer excluding bromophenol blue as described [25]. Protein concentration was determined using the Bio-Rad Dc Protein Assay Reagents. Bromophenol blue was then added and 50 µg of protein in each lane were separated by SDS-10% PAGE and transferred to nitrocellulose membranes. Membranes were probed with indicated antibodies at 4°C overnight and with secondary antibodies conjugated with horseradish peroxidase (HRP, Amersham), followed by the ECL detection reagents (Thermo Scientific). Anti-merlin (Santa Cruz), -actin (NEOMARKERS), -MST1/2, and –phospho-MST1/2 (Cell Signaling) antibodies were used.

### Lenti- and Retro-virus Transduction

To knockdown merlin expression, M19-MEL and WM1552C cells were transduced following the manufacturer’s directions with the lentiviruses carrying shRNA constructs against human merlin or a non targeting (NT) shRNA control (Open Biosystems, Addgene). Full length merlin isoform I with a COOH-terminal v5 epitope tag was cloned into a retroviral expression vector, pQCXIP (BD Sciences). The expression construct was verified by DNA sequencing. The infected cells were selected in the complete culture media containing puromycin. Subsequent experiments were carried out using the pooled populations of puromycin-resistance cells after drug selections.

### Quantitative Real-time PCR

mRNAs were isolated from the indicated cells using the Qiagen RNeasy mini kit. 5 µg of mRNA was used to create cDNA using reverse transcriptase III (Invitorgen). Real-time PCR reaction was carried out using FastStart SYBR Green Master Mix (Roche Diagnostics) in a Stratagene Mx3005P real time machine as detailed [25]. The data was analyzed using MxPro Software and GAPDH was used as a control for comparison.

### Proliferation Assay

Melanoma cells were counted by a Beckman Coulter Particle Counter Z1 and 2000 cells per well were plated in 96 well plates in triplicate. Cell proliferation assays were performed using Premix WST1 kit (TaKaRa) following the manufacturer’s instruction. Post confluent cell proliferation was performed by plating 100,000 cells per well in 24 well plates, and at the indicated time points, cells were trypsinized and counted using a Beckman Coulter Particle Counter Z1.

### Invasion and Migration Assay

Transwell migration/invasion assays were carried out as previously described [25]. Briefly, cells were trypsinized and re-suspended in media with or without 1% FBS (for M19-MEL and MeWo) or insulin (for WM1552C) cells at 1×10^6^ cells/ml. Cells were placed in the upper chambers of Transwell inserts (Costar) coated with (invasion assay) or without (migration assay) Matrigel (BD Bioscience). Complete media was placed in the bottom of each well. Cells were then incubated at 37 degrees for 20 (for migration assay) or 30 (for invasion assay) hours. Cells migrated or invaded to the underside of the inserts were then fixed and stained using a Diff-Quick Stain Set (Siemens). The underside cells in 15 randomly selected 200X microscopic fields were then countered using the QCapture Imaging Software.

### Soft Agar Colony Formation Assay

A base layer of 0.7% soft agar was applied to 6-well tissue culture plates. After solidification, a layer of 0.35% soft agar containing 25,000 cells/ml was applied on top of the base layers. Cells were allowed to grow for three weeks in a 5% CO_2_ humidified incubator. Pictures were taken in 15 randomly selected 50× microscopic fields and colonies were counted in these fields. Bars represent mean number of colonies per field +/− the SD.

### Subcutaneous Melanoma Growth Experiments and Immunohistochemistry (IHC)

All animal procedures were performed according to NIH guidelines and approved by the Institutional Animal Care and Use Committee of the Mount Sinai School of Medicine. Melanoma Cells were suspended in the Hanks Balanced Salt Solution (Invitrogen). 5×10^6^ cells of transduced M19-MEL cells were injected into each of 11 immunocompromised B6.129S7-Rag1^tm1/MOM^ mice (Jackson Laboratories). Tumors were allowed to grow for six weeks and at the end of six weeks the mice were assessed for palpable tumors and recorded. 10×10^6^ cells and 1×10^6^ cells of the transduced WM1552C and transduced MeWo cells, respectively, were injected into each immunocompromised Rag2/II2rg mice (Taconic). 6–8 mice were injected with each of the transduced melanoma cells as detailed in the figure legends. Tumors were allowed to grow for indicated time and the tumor volumes were measured at the indicated intervals as previously described [25], [51]. At end of the experiments, the tumors were excised and divided into two groups. Portions of the tumors were frozen at −80 degrees C for analyzing merlin protein levels by Western blots and others were fixed and embedded in paraffin for immunohistochemical analysis as previously described [25], [51].

### H_2_0_2_ Treatment of the Melanoma Cells

Transduced melanoma cells were plated in 6-well plates and allowed to grow for 24 h in complete RPMI media. Indicated amounts of H_2_0_2_ (Fisher) as detailed in the figure legends were added to serum-free RPMI media for indicated period at 37 degrees. These cells were then lysed in 4X SDS sample buffer without bromophenol blue, and subsequent western blot analysis was performed as described [25].

### Statistical Analyses

Chi Squared test was performed to determine the significance of tumor incidence in the subcutaneous melanoma growth experiment. All other results were analyzed using the student T-test. A *p*-value of <0.05 was considered statistically significant.

## Supporting Information

Figure S1 **Merlin knockdown enhances melanoma cell motility.** **A**, Transwell Migration assay of WM1552C cells transduced with shRNA targeting merlin (sh#6 and sh#11) or non-targeting shRNA (shNT), Bars represent the mean migrated cells in 15 randomly selected 200X microscopic fields. *denotes a *p*-value <0.01. **B**, Transwell Migration assay of M19-MEL cells transduced with shRNA targeting merlin (sh#2 and sh#4) or non- targeting shRNA (shNT), Bars represent the mean migrated cells in 15 randomly selected 200X microscopic fields. *denotes a *p*-value <0.01,
(TIF)

Figure S2 **Merlin expression is decreased in established subcutaneous melanomas derived from WM1552C cells with or without merlin knockdown.** Representative merlin immunoreactivity on the sections derived from human benign nevi (A–B), MeWo Mer subcutaneous tumors expressing exogenous merlin (C–D), or subcutaneous melanomas derived from WM1552C cells transduced with shRNA targeting merlin (sh #6 and sh#11, E–G) or control shRNA (shNT, H). The sections (A, C, E, G, and H) were reacted to an anti-merlin antibody (Santa Cruz) or a secondary antibody only (B, D, and F). Bar, 200 µm in A–B and 100 µm in C–H.
(TIF)

Figure S3 **Endogenous merlin expression is diminished in a subset of human melanoma cell lines.** Western blot analysis of endogenous merlin expression was performed using the indicated human melanoma cell lysates and anti-merlin antibody (Santa Cruz). Arrows indicate metastatic melanoma cell lines with little or significantly reduced merlin expression. 50 µg of protein was loaded in each lane and actin was used as a loading control.
(TIF)

Figure S4 **Increasing FBS concentration in cell culture media does not affect endogenous merlin protein levels.** Western blot analysis of endogenous merlin expression in WM1552C human melanoma cells grown in recommended MCDB media supplemented with 2% or 10% FBS, or in RPMI media with 10% FBS for 72 h. 50 µg of protein was loaded in each lane and actin was used as a loading control
(TIF)

Table S1 **Merlin knockdown confers tumorigenicity to M19-MEL human melanoma cells.** Table shows the tumor incidences in Rag1 mice sixty days post injection of 5×10^6^ of M19-Mel cells with or without merlin knockdown.
(TIF)
