# Supplementary figures and images for: Merlin Is a Negative Regulator of Human Melanoma Growth
Source: PLoS One. 2012 Aug 17;7(8):e43295. doi: 10.1371/journal.pone.0043295 (PMC3422319; doi:10.1371/journal.pone.0043295)

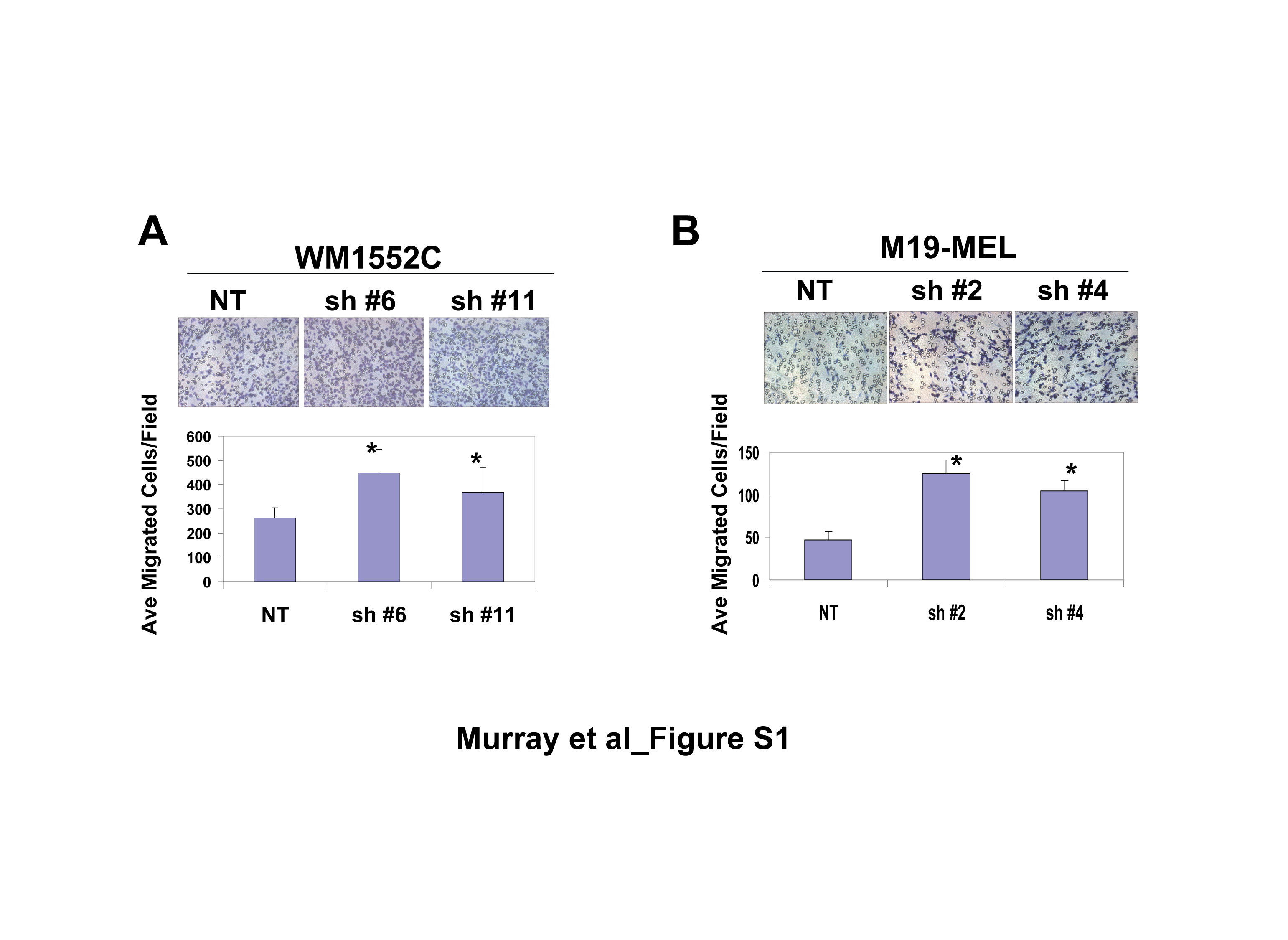

Supplement: Figure S1 — Merlin knockdown enhances melanoma cell motility. A, Transwell Migration assay of WM1552C cells transduced with shRNA targeting merlin (sh#6 and sh#11) or non-targeting shRNA (shNT), Bars represent the mean migrated cells in 15 randomly selected 200X microscopic fields. *denotes a p-value <0.01. B, Transwell Migration assay of M19-MEL cells transduced with shRNA targeting merlin (sh#2 and sh#4) or non- targeting shRNA (shNT), Bars represent the mean migrated cells in 15 randomly selected 200X microscopic fields. *denotes a p-value <0.01, (TIF) [file pone.0043295.s001.tif]

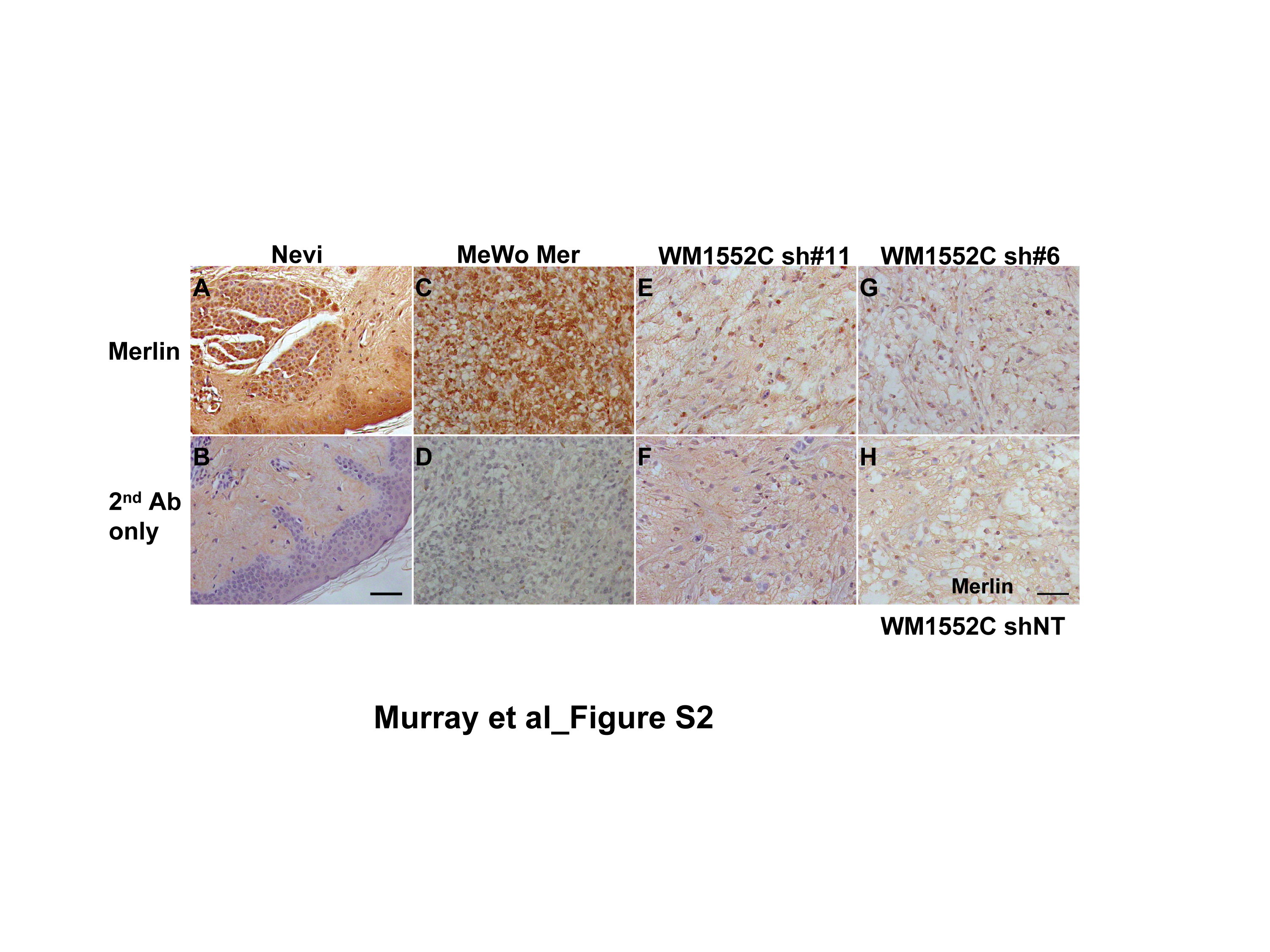

Supplement: Figure S2 — Merlin expression is decreased in established subcutaneous melanomas derived from WM1552C cells with or without merlin knockdown. Representative merlin immunoreactivity on the sections derived from human benign nevi (A–B), MeWo Mer subcutaneous tumors expressing exogenous merlin (C–D), or subcutaneous melanomas derived from WM1552C cells transduced with shRNA targeting merlin (sh #6 and sh#11, E–G) or control shRNA (shNT, H). The sections (A, C, E, G, and H) were reacted to an anti-merlin antibody (Santa Cruz) or a secondary antibody only (B, D, and F). Bar, 200 µm in A–B and 100 µm in C–H. (TIF) [file pone.0043295.s002.tif]

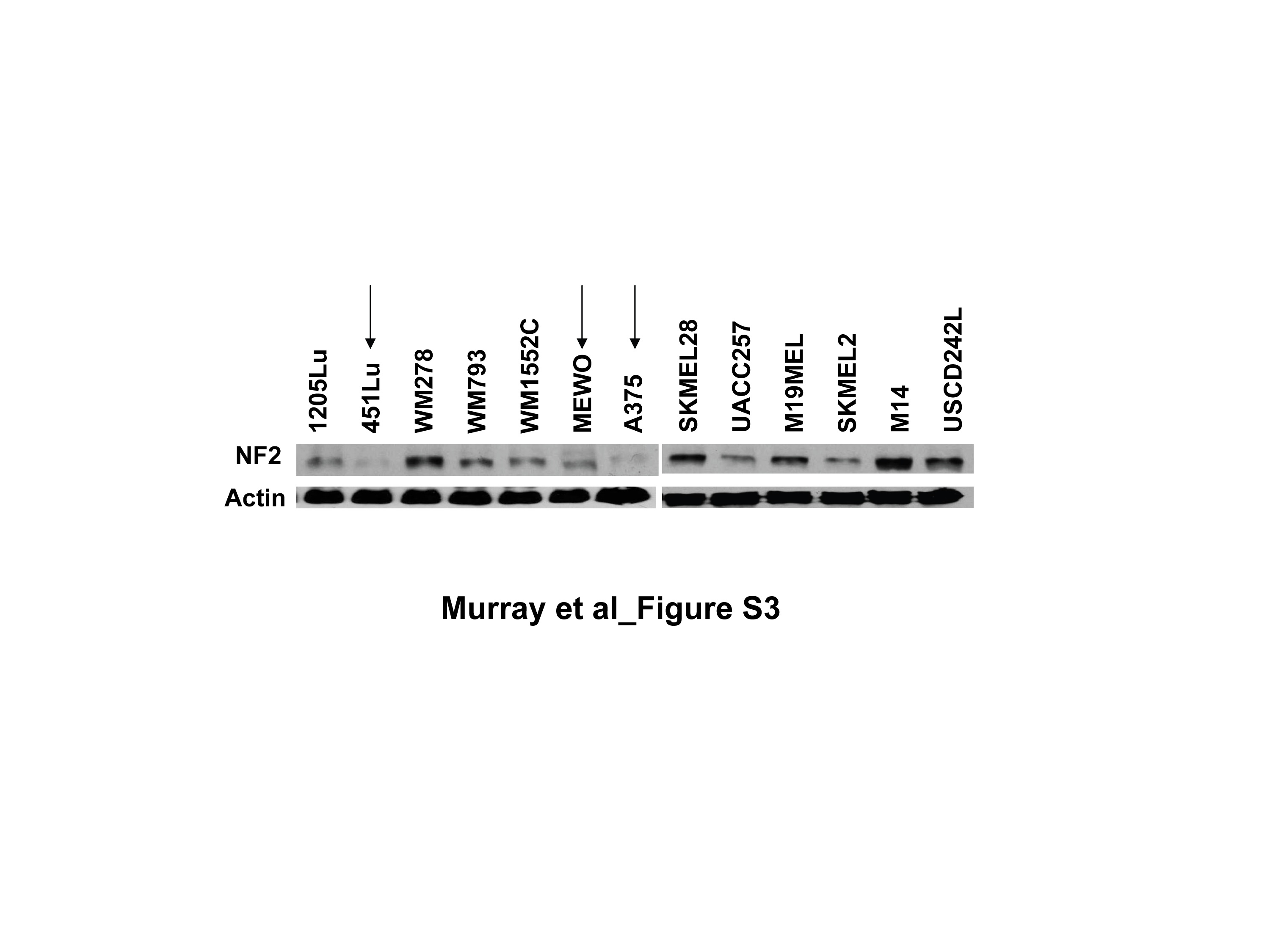

Supplement: Figure S3 — Endogenous merlin expression is diminished in a subset of human melanoma cell lines. Western blot analysis of endogenous merlin expression was performed using the indicated human melanoma cell lysates and anti-merlin antibody (Santa Cruz). Arrows indicate metastatic melanoma cell lines with little or significantly reduced merlin expression. 50 µg of protein was loaded in each lane and actin was used as a loading control. (TIF) [file pone.0043295.s003.tif]

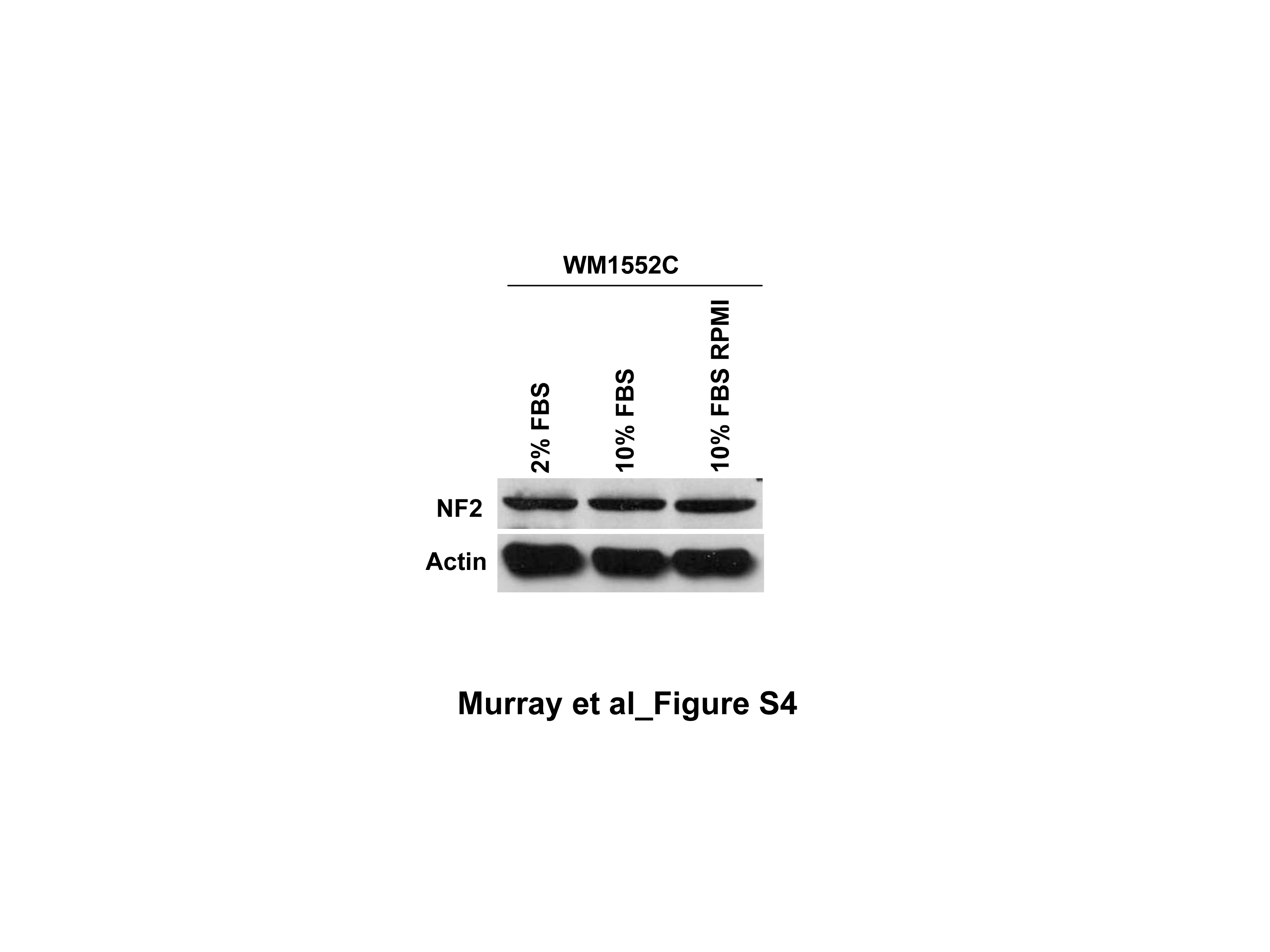

Supplement: Figure S4 — Increasing FBS concentration in cell culture media does not affect endogenous merlin protein levels. Western blot analysis of endogenous merlin expression in WM1552C human melanoma cells grown in recommended MCDB media supplemented with 2% or 10% FBS, or in RPMI media with 10% FBS for 72 h. 50 µg of protein was loaded in each lane and actin was used as a loading control (TIF) [file pone.0043295.s004.tif]

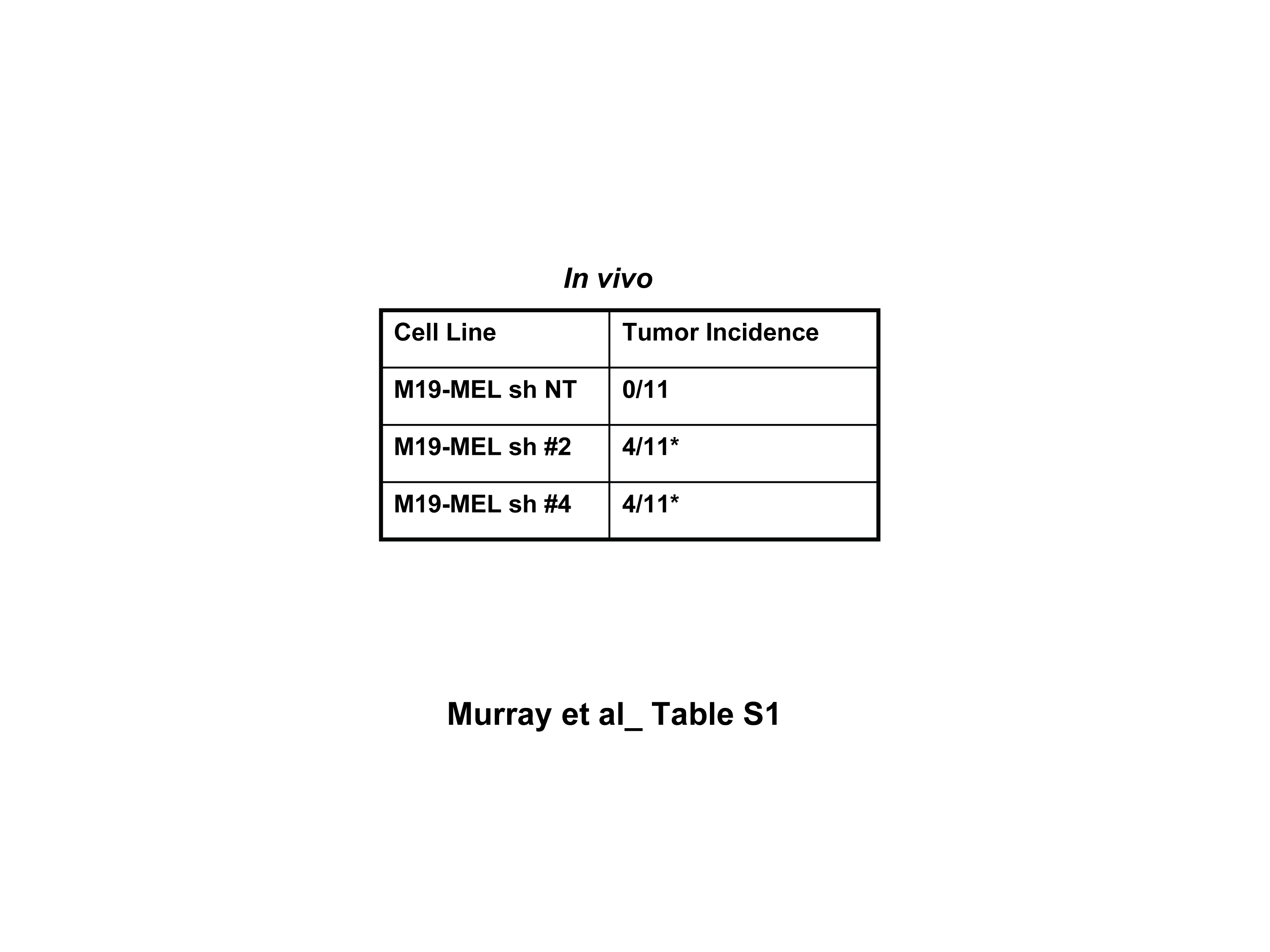

Supplement: Table S1 — Merlin knockdown confers tumorigenicity to M19-MEL human melanoma cells. Table shows the tumor incidences in Rag1 mice sixty days post injection of 5×10^6^ of M19-Mel cells with or without merlin knockdown. (TIF) [file pone.0043295.s005.tif]
